# A Fuzzy Radial Basis Adaptive Inference Network and Its Application to Time-Varying Signal Classification

**DOI:** 10.1155/2021/5528291

**Published:** 2021-06-23

**Authors:** Long Huang, Shaohua Xu, Kun Liu, Ruiping Yang, Lu Wu

**Affiliations:** ^1^College of Computer Science and Engineering, Shandong University of Science and Technology, Qingdao 266590, Shandong Province, China; ^2^Shandong Computer Science Center (National Supercomputer Center in Jinan), Jinan 250014, Shandong Province, China

## Abstract

A fuzzy radial basis adaptive inference network (FRBAIN) is proposed for multichannel time-varying signal fusion analysis and feature knowledge embedding. The model which combines the prior signal feature embedding mechanism of the radial basis kernel function with the rule-based logic inference ability of fuzzy system is composed of a multichannel time-varying signal input layer, a radial basis fuzzification layer, a rule layer, a regularization layer, and a T-S fuzzy classifier layer. The dynamic fuzzy clustering algorithm was used to divide the sample set pattern class into several subclasses with similar features. The fuzzy radial basis neurons (FRBNs) were defined and used as parameterized membership functions, and typical feature samples of each pattern subclass were used as kernel centers of the FRBN to realize the embedding of the diverse prior feature knowledge and the fuzzification of the input signals. According to the signal categories of FRBN kernel centers, nodes in the rule layer were selectively connected with nodes in the FRBN layer. A fuzzy multiplication operation was used to achieve synthesis of pattern class membership information and establishment of fuzzy inference rules. The excitation intensity of each rule was used as the input of T-S fuzzy classifier to classify the input signals. The FRBAIN can adaptively establish fuzzy set membership functions, fuzzy inference, and classification rules based on the learning of sample set, realize structural and data constraints of the model, and improve the modeling properties of imbalanced datasets. In this paper, the properties of FRBAIN were analyzed and a comprehensive learning algorithm was established. Experimental validation was performed with classification diagnoses from four complex cardiovascular diseases based on 12-lead ECG signals. Results demonstrated that, in the case of small-scale imbalanced datasets, the proposed method significantly improved both classification accuracy and generalizability comparing with other methods in the experiment.

## 1. Introduction

Signal analysis in nonlinear dynamic systems is an active area of research in the fields of artificial intelligence and data modeling [1]. However, due to noise in the measuring instrument or environment, the information obtained by sensors may be inaccurate or incomplete. Therefore, fuzzification analysis is usually needed for this kind of signal [2]. In addition, for some complex time-varying systems, it is difficult to obtain large-scale or complete signal sets due to the nonrepeatability of state process or the high cost of signal acquisition. Deep neural network models have been applied to the classification of time-varying signals, including dynamic recurrent neural networks [3–5], deep recursive networks [6], long short-term memory (LSTM) models [7], and deep convolutional neural networks [8–10]. However, these models depend on the completeness of training datasets and are not suitable for the time-varying signal classification of incomplete or ambiguous datasets. Therefore, the application of signal pattern classification and state prediction in complex nonlinear systems still faces some challenges.

Fuzzy neural networks can be effective for incomplete and inaccurate process information modeling and analysis [11]. Several algorithms, combining neural networks and fuzzy processing, have been proposed for signal analysis. Uyar and İlhan propose a genetic algorithm based trained recurrent fuzzy neural networks for the diagnosis of heart diseases [12]. Fei and Wang proposed an adaptive fuzzy neural network control scheme based on a radial basis neural network to enhance the performance of a shunt active power filter [13]. Camastra et al. presented a fuzzy decision system for environmental risk assessments of genetically modified plants, based on a Mamdani inference [14]. Liu et al. proposed a generalized prediction system called a recurrent self-evolving fuzzy neural network (RSEFNN), which utilized online gradient descent learning rules to classify driving fatigue in EEG regression problems [15]. Nazari et al. proposed a fuzzy inference, fuzzy analytic, and hierarchy process-based clinical decision support system for the diagnosis of heart disease. The corresponding fuzzy inference rules were acquired using expert knowledge [16]. Ilbahar et al. proposed a novel approach to risk assessment for occupational health, based on the Pythagorean fuzzy analytic hierarchy process and a fuzzy inference system [17]. Mohamad and Mukhtar developed a weighted Mamdani-type fuzzy inference model for a relative ideal preference system, based on fuzzy if-then rules [18].

A comprehensive analysis shows that most of existing fuzzy neural networks are the fuzzy analysis models based on fuzzy feature extraction and if-then rules. These techniques can be regarded as “static models” and are based on fuzzy logic that requires embedded expert knowledge. In the fuzzy analysis of time-varying signals, fuzzy inference processes are primarily constructed using backpropagation and circulation [19]. As a result, these methods exhibit limitations, such as weak adaptive learning capabilities, low efficiency for large-scale dataset processing, or professional experience requirements.

The radial basis neural network (RBNN) is a widely used kernel function technique [20]. It achieves nonlinear mapping by varying the parameters of nonlinear neuron transformation functions and improves learning speeds of network by linearizing the connection weight adjustments. It offers the advantages of fewer model parameters and low computational complexity and can form effective feature interfaces [21, 22]. Xu and He extended the processing domain of a radial basis neural network to the time dimension and proposed a radial basis process neural network (RBPNN) model [23]. This algorithm accepts multichannel time-varying signals as input and can embed distribution characteristics for typical signal samples, based on radial basis kernel center functions. However, it exhibits a shallow structure with low information capacity and includes strict requirements for sample set completeness. A novel fuzzy neural network can be established when rule-based reasoning for fuzzy logic systems is combined with the feature knowledge embedding mechanism and learning properties of an RBPNN. This provides a new methodology for the fuzzy classification of time-varying signals. Fuzzy neural network also has important applications in the field of robust adaptive control. Kong et al. proposed an adaptive fuzzy neural network control scheme using impedance learning for the multiple constrained robots with unknown dynamics and time-varying constraints, which improved the environment-robot interaction [24]. He et al. designed a boundary control method based on bionics to control a two-link rigid-flexible wing, which effectively improves the mobility and the flexibility of aircraft [25]. He et al. used radial basis function neural network to approximate the aerodynamic perturbation torque and proposed a hierarchical control scheme to study the trajectory tracking problem of microaerial vehicles in the longitudinal plane, and it is shown that the tracking errors are bounded [26].

In this paper, a novel fuzzy radial basis adaptive inference network (FRBAIN) is proposed for multichannel time-varying signal classification. First, the radial basis process neural network (RBPNN) was fuzzified to establish a fuzzy radial basis process neural network (FRBPNN), composed of a time-varying signal input layer, a fuzzy radial basis kernel transformation layer, and a membership degree output layer. The dynamic time warping (DTW) algorithm, which is insensitive to the contraction and expansion of time-varying signals, was used to measure the similarity between time-varying signal distribution features. The dynamic fuzzy C-means clustering (DFCM) algorithm was used to divide the sample set pattern classes into subclasses with similar features. Typical characteristic signal samples for each pattern subclass could be determined, which were used as the kernel centers of radial basis process neurons (RBPNs). The exponential sigmoid function with fuzzy membership properties was used as the activation function, to produce the output of each RBPN based on the fuzzy set membership degree and the fuzzification of RBPN. In this case, the fuzzy radial basis process neurons (FRBPNs) become the parameterized membership functions, relative to the fuzzy set of the pattern subclass. Nodes in the fuzzy rule layer were selectively connected with nodes in FRBN layer according to the signal categories of FRBN kernel centers. A fuzzy multiplication operation was then used to synthesize membership information of fuzzy sets and establish fuzzy reasoning rules. The output of rule layer was regularized and normalized as the excitation intensity. A T-S fuzzy function was used as the classifier, which accepted the excitation intensity of each rule as input to classify multichannel time-varying input signals.

The FRBAIN proposed in this paper can realize the embedding of the time-varying signal each pattern class diversity prior feature knowledge, as well as the structure and data constraints of the model. Through the learning of the sample set, it can adaptively establish the fuzzy inference rules and classification rules with fine-grained and effectively improve the problem that the features of the pattern classes with few samples in the small-scale, and imbalanced dataset are suppressed and weakened in the training, and the robustness and generalization ability of the model are improved.

Cardiovascular disease diagnosis based on ECG signal is a typical multichannel signal classification problem. ECG signals exhibit nonstationarity, irregular periods, stretch drift, and high background noise, resulting in fuzziness and multiple solutions [27]. In 12-lead ECG signals, atrial premature beat, frequent ventricular premature beat, atrial tachycardia, and atrial fibrillation with rapid ventricular rate exhibit similar distributions and complex combination characteristics. In this study, FRBAIN was used to classify and diagnose these four diseases using small-scale and imbalanced datasets, to verify the feasibility and effectiveness of the proposed model.

The remainder of this paper is organized as follows. After discussing the challenges of neural network-based time-varying signal classification, in Section 2, the theoretical framework for the proposed model is given. A comprehensive learning algorithm for the FRBAIN is proposed in Section 3. In Section 4, the classification experiment of ECG signals and result analysis are carried out. In Section 5, the work of this paper is summarized, and the advantages, limitations, and potential applications of this method are pointed out.

## 2. A Dynamic Fuzzy Radial Basis Adaptive Inference Network

### 2.1. A Fuzzy Radial Basis Neural Network

**Figure 1:**
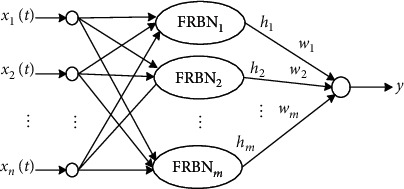
The FRBFN structure.

The information processing domain in RBPNN was extended to fuzzy sets to establish a fuzzy radial basis neural network (FRBNN). This model consists of a multichannel time-varying signal input layer, a radial basis fuzzification layer, and an output layer, as shown in Figure 1.

In the figure, *X*(*t*)=(*x*_1_(*t*), *x*_2_(*t*),…, *x*_*n*_(*t*))(*t* ∈ [0, *T*]) is a multichannel time-varying input signal, FRBN_*j*_(*j*=1,2,…, *m*) denotes fuzzy radial basis neurons (FRBNs), and *h*_*j*_ is the *j*^th^ output of the FRBN. The term *w*_*j*_ is the connected weight between the hidden layer and the network output unit, and *y* is the output of network. The input signal vector *X*(*t*) can be linearly transferred to the FRBN layer. Information fusion and the fuzzification processing of multichannel input signals were achieved in the FRBN layer, in addition to membership degree output. The fuzzy classification of input signals was performed in the output unit.

The radial basis kernel function was assumed to be an exponential sigmoid with fuzzy membership [28]. The output of the *j*^th^ FRBN is then given by

(1) $$\begin{array}{c} h_{j}\left(X\left(t\right)\right)=\frac{1}{1+\exp\left(-a\left(-d_{v}^{2}\left(X\left(t\right),{\tilde{Z}}_{j}\left(t\right)\right)/\sigma^{2}\right)-c\right)}, \end{array}$$

where ${\tilde{Z}}_{j}\left(t\right)$ is the kernel center vector in the *j*^th^ FRBN. The term $d_{v}\left(X\left(t\right),{\tilde{Z}}_{j}\left(t\right)\right)$ represents the distance (or fuzzy feature similarity) between *X*(*t*) and ${\tilde{Z}}_{j}\left(t\right)$, based on a certain norm, and *σ* > 0 is a smoothing parameter. *a* and *c* are morphological parameters. The FRBNN output is a fuzzy linear weighted sum of the hidden layer node outputs. It can be calculated as follows:

(2) $$\begin{array}{c} \tilde{y}=F\left(\tilde{X}\left(t\right)\right)=\sum_{j=1}^{m}{\tilde{w}}_{j}\cdot h_{j}\left(\tilde{X}\left(t\right)\right). \end{array}$$

### 2.2. The Fuzzy Radial Basis Adaptive Inference Network

#### 2.2.1. The FRBAIN Model

**Figure 2:**
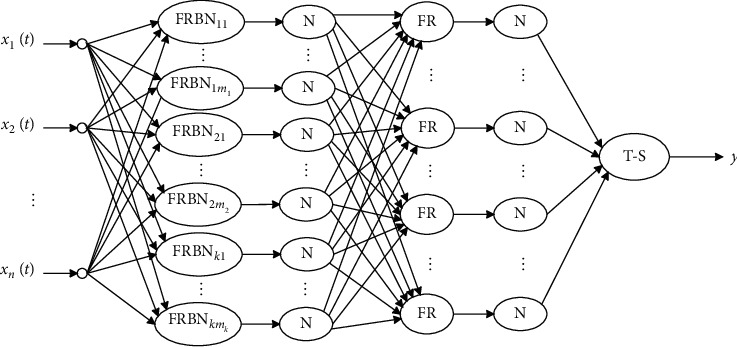
The fuzzy radial basis adaptive inference network.

The DFRBAIN is composed of a multichannel time-varying signal input layer, a radial basis fuzzification layer, regularization layer I, a fuzzy rule layer, regularization layer II, and a T-S fuzzy classifier. This structure is shown in Figure 2, where *x*_*i*_(*t*)(*i*=1,2,…, *n*) is the multichannel time-varying input signal, and FRBN_*klk*_(*k*=1,2,…, *K*; *l*_*k*_=1,2,…, *m*_*k*_) corresponds to the *l*^th^ subclass in the *k*^th^ pattern class. *m*_*k*_ is the number of pattern subclasses in the *k*^th^ pattern class. The FR terms are nodes in the fuzzy rule layer units and T-S is a fuzzy classifier.

The following mapping relationships between the input and output of each FRBAIN layer can be determined from Figure 2.

1. The input layer accepts a multichannel time-varying signal *X*(*t*)=(*x*_1_(*t*), *x*_2_(*t*),…, *x*_*n*_(*t*))(*t* ∈ [0, *T*].
2. In the radial basis fuzzification layer, FRBNs are used as the fuzzy set membership functions and exponential sigmoid is used to represent the radial basis kernel function. The output of *X*(*t*) at the *j*^th^ node in this layer can be represented as follows:

(3) $$\begin{array}{c} \mu_{A_{j}}\left(X\left(t\right)\right)=\frac{1}{1+\exp\left(-a\left(-d_{v}^{2}\left(X\left(t\right),{\tilde{Z}}_{j}\left(t\right)\right)/\sigma_{j}^{2}\right)-c\right)}, \end{array}$$

where *A*_*j*_ is the universal fuzzy set, *μ*_*A*_*j*__ is the membership function for *A*_*j*_, ${\tilde{Z}}_{j}\left(t\right)$ represents the kernel center signal vector, *σ*_*j*_ is an FRBN smoothing parameter, and *j*=1,2,…, *n*_*i*_. *n*_*i*_ is the number of samples in the *i*^th^ pattern subclass.

Kernel center functions for the fuzzy radial basis neurons were determined using the following approach.

(1) Multichannel time-varying signal sample sets, containing *K* pattern classes, were used as input. The DTW algorithm [29], which is insensitive to contraction and extension of time-varying signals, was used to measure the similarity between signal sample features. The DFCM clustering algorithm [30] was then used to divide the samples from each pattern class into subclasses exhibiting similar characteristics, before selecting typical samples from each. The *k*^th^ pattern class was divided into *m*_*k*_(*k*=1,2,…, *K*) partitions and the sample set containing a total of *m*=∑_*k*=1_^*K*^*m*_*k*_ pattern subclasses.
(2) The selected *m* typical signal samples were arranged as follows: *Z*_11_(*t*),…, *Z*_1*m*_1__(*t*), *Z*_21_(*t*),…, *Z*_2*m*_2__(*t*),…, *Z*_*K*1_(*t*),…, *Z*_*Km*_*K*__(*t*). Here, the first subscript of *Z*_*kl*_(*t*)(*l*=1,2,…, *m*_*k*_; *k*=1,2,…, *K*) indicates the cluster center category and the second subscript represents the *l*^th^ cluster center of the *k*^th^ pattern class. The *Z*_*kl*_(*t*) terms were sequentially assigned as kernel centers for each node in the RBFPN layer, with *m* total nodes in the FRBN. Structural and data constraints were then produced through the embedding of prior feature knowledge.
(3) Regularization layer I normalized the outputs of FRBN layers, which is defined as
  (4) $$\begin{array}{c} \mu_{A_{ij}}^{\prime }\left({\tilde{X}}_{i}\right)=\frac{\mu_{A_{ij}}\left(X_{i}\right)}{\sum_{j=1}^{n_{i}}\mu_{A_{ij}}\left(X_{i}\right)}, \end{array}$$
where $\mu_{A_{ij}}^{\prime }\left({\tilde{X}}_{i}\right)$ represents the regularized output of *μ*_*A*_*ij*__(*X*_*i*_). The FRBNs were used as parameterized membership functions and the membership degree of the input signal, relative to the fuzzy set, was adaptively determined by learning the instance sample set.
(4) The fuzzy rule layer connects the antecedent (regularization nodes) and the conclusion nodes (FR output nodes). Connection rules required that each rule node was connected only to a regular node from each input (after being fuzzed). This process is shown in Figure 2 for the connection between the third and fourth layers. In the classification problem, the fuzzy sets corresponding to the pattern subclasses were the same as the pattern classes. In the *K*-classification problem, the number of fuzzy sets was denoted by *K*. Since the FRBN layer outputs are according to the pattern subclass fuzzy set, the number of nodes in the rule layer is given by
  (5) $$\begin{array}{c} L=K^{m}. \end{array}$$
In practice, connection rules and connection methods may differ and the number of nodes and generation rules in the rule layer will vary. Using fuzzy multiplication, the output of the *k*^th^ rule node can be expressed as follows:
  (6) $$\begin{array}{c} z_{k}=\prod_{i=1}^{n}\mu_{A_{ik}}^{\prime }\left({\tilde{X}}_{i}\right), \end{array}$$
where other *T*-normal operators that perform fuzzy “and” operations can also be used in fuzzy multiplication.
(5) Regularization layer II processes outputs of the fuzzy rule layer. The output of the *l*^th^ node in this layer can be considered the activation intensity of the *l*^th^ rule.
(6) The T-S fuzzy classifier accepts the *L* normalized rule activation intensity *q*_1_, *q*_2_,…, *q*_*L*_, output by regularization layer II, as input. The output of the T-S fuzzy classifier is then given by
  (7) $$\begin{array}{c} \tilde{y}=\tilde{f}\left(\sum_{k=1}^{L}\left({\tilde{w}}_{k}q_{k}+r_{k}\right)\right), \end{array}$$
where $\tilde{f}$ is the activation function for the classifier and ${\tilde{w}}_{k}$ and *r*_*k*_ are classifier parameters.

#### 2.2.2. The Extended FRBAIN Model

**Figure 3:**
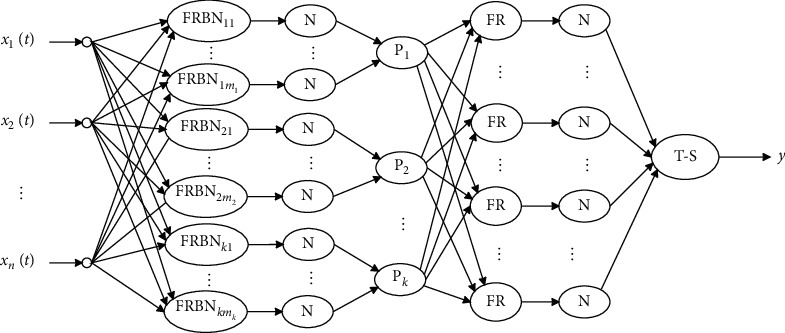
The extended FRBAIN model.

As seen in (5), an increase in the number of nodes in the FRBN layer will cause an exponential increase in the number of nodes in the rule layer. To solve this problem, an extended FRBAIN (E-FRBAIN) model was constructed by adding a pattern layer between the FRBN and rule layers, representing the membership degree of pattern class fuzzy sets, as shown in Figure 3.

In Figure 3, each node in the FRBN layer converges the output membership degree to the corresponding node *P*_*k*_(*k*=1,2,…, *K*) in the pattern layer, according to pattern subclass labels for the kernel center and containment relationships with the pattern class. The output of each node in the pattern layer can be calculated using a “sum” or “maximum” operation.

The output of the pattern layer is then given by

(8) $$\begin{array}{c} q_{k}=\sum_{j\in \Omega_{k}}h_{j}\left(k=1,2,\ldots ,K\right). \end{array}$$

In (8), *h*_*j*_ is the regularized output of the *j*^th^ FRBN and *K* is the number of signal sample pattern classes contained in the training set. The term Ω_*k*_ is the serial number set for the FRBN layer node corresponding to the *k*^th^ pattern class.

In the classification problem, the fuzzy set corresponding to the pattern class is the same as the fuzzy set corresponding to the pattern subclass. Equation (8) suggests the fuzzy membership degree for each node in the pattern layer integrates membership degree information for each pattern subclass in the fuzzy set. The number of fuzzy sets in the *K*-classification problem is denoted by *K*. Multiplication rules require the number of nodes in the rule layer to be *L*=*K*^*K*^, where *K* ≪ *m* in practice. Therefore, the E-FRBAIN model effectively reduces the number of nodes in the rule layer, while generated fuzzy rules simultaneously retain membership degree information for pattern subclasses.

#### 2.2.3. Property Analysis

Comprehensive analysis shows that the properties of the FRBAIN are as follows:

1. In this paper, using an algorithm that combines DTW and DCFM, each pattern samples of the dataset are divided into pattern subclasses with more similar features, and the diversity typical features samples of each pattern subclass are determined, which are used as the kernel centers of the RBPN. When the number of typical feature signal samples is determined, the number of nodes in the radial basis fuzzy layer (the first hidden layer) in the model is also determined. The number of nodes from the second hidden layer to the final classification unit of the network model is calculated according to the fuzzy inference rules, which realizes the structural constraints of the model.
2. The typical signal samples of each pattern subclasses are used as the radial basis kernel centers, which implicitly expresses the category features of each pattern signal, realizes the memory and storage of the typical signal distribution features of each pattern class, and strengthens the role of prior feature knowledge in classification. In the fuzzy radial basis kernel transformation layer, the input signals and the kernel centers are measured for feature similarity, and the transformations of the node units in the subsequent each layer are calculated according to the output of the fuzzy radial basis neuron layer, which realizes the data constraints of the model.
3. In this paper, the typical signal feature samples of each pattern class are used as the kernel centers of FRBNs, which can improve the phenomenon that the features of the pattern class with less samples in the imbalanced dataset are suppressed and weakened in the training and reduce the optimization search space of model parameters. Moreover, the model proposed in this paper contains only a few parameters, and the parameters can be determined adaptively through the learning of small-scale dataset, which has a strong ability of signal sample feature identification. It is suitable for the modeling and analysis of small-scale imbalanced datasets in mechanism and can improve the robustness and generalization ability of the model.

#### 2.2.4. Algorithm Complexity

For the FRBAIN model proposed in this paper, assuming that the number of samples in the training dataset is *N*, the number of nodes in the radial basis fuzzification layer is *L*, the number of nodes in the fuzzy rule layer is *m*, and the number of pattern classes is *K*, then the time complexity of DTW algorithm, the radial basis fuzzification layer, the pattern layer, the fuzzy rule layer, and the T-S fuzzy classifier are *O*(*N*^2^), *O*(*L*), *O*(*L* × *K*), *O*(*K*^*m*^), and *O*(*m* × *m*), respectively. Adding all the items together, the total time complexity of the proposed method is *O*(*N*^2^)+*O*(*L*)+*O*(*L* × *K*)+*O*(*K*^*m*^)+*O*(*m* × *m*).

## 3. The Learning Algorithm

The FRBAIN learning process can be divided into 3 stages. (1) The DTW algorithm can be used to measure the similarity between signal sample features. The DFCM algorithm can then be used to divide pattern classes for the training set into several subclasses, identifying typical feature signal samples in each subclasses. (2) The total number of pattern subclasses is then set to the number of nodes in the FRBN layer. Typical signal samples are then used as kernel centers for each FRBN while calculating the output. (3) The gradient descent algorithm is used to train the FRBAIN parameters.

### 3.1. The DTW Algorithm

DTW is a similarity measurement technique for time-series signal distribution characteristics, based on dynamic programming, which combines distance calculations and time warping [31]. The algorithm requires an optimal time warping function *M*=∅(*N*), which nonlinearly maps the time axis of time-series signals to the time axis of a reference template. The resulting function satisfies

(9) $$\begin{array}{c} D=\min\sum_{n=1}^{N}d\left\{T\left(n\right),R\left[\emptyset \left(n\right)\right]\right\}. \end{array}$$

It is assumed the test template includes an *N*-frame feature vector, the reference template includes an *M*-frame feature vector, and *d*{*T*(*n*), *R*[∅(*n*)]} is the distance measurement between the *n*^th^ frame feature vector *T*(*n*) in the test template and the *m*^th^ frame feature vector *R*(*m*) in the reference template. The term *D* is a warping function representing the minimum cumulative distance for each frame of the test and reference templates under optimal time warping. Smaller values indicate higher similarity between two signal distribution features. The primary steps in the DTW algorithm are as follows [32]:

 
*Step 1*. A signal sequence contrast matrix is constructed.
 
*Step 2*. The distance measure and warping cost functions are defined.
 
*Step 3*. A warping path is determined using a dynamic programming algorithm.
 
*Step 4*. The optimal path is identified and the similarity degree between signal sequences is calculated.

### 3.2. The Dynamic Fuzzy C-Means Clustering Algorithm

The DFCM clustering algorithm is a dataset partitioning technique that acquires membership degree information from each sample point for all cluster centers, through optimization of the objective function, prior to determining sample point classes [33]. The coupling and separation degrees between signal samples are then calculated by setting different clustering numbers, evaluating the corresponding results, and selecting the optimal clustering output.

Suppose the sample set contains *N* signals and *c* clusters. The coupling degree *C*_*d*_(*c*) representing the in-class compactness and the separation degree *S*_*d*_(*c*), reflecting the between-class separation [34]. The following formula was used to evaluate the clustering results:

(10) $$\begin{array}{c} G\;D\left(c\right)=\alpha C_{d}\left(c\right)+\left(1-\alpha \right)\frac{1}{S_{d}\left(c\right)}, \end{array}$$

where *α* is the coupling weight factor. Smaller *G*  *D*(*c*) values represent better clustering results, and the *C* value corresponding to the minimum of *G*  *D*(*c*) is the optimal number of clusters. Partitions of the sample set produce the best clustering results.

### 3.3. The Training of FRBAIN

The parameters of FRBAIN include the radial basis kernel center smoothing parameter vector *σ*, the morphological parameters *a* and *b*, the connection weight matrix *W* (from the rule layer to the fuzzy classifier), and the parameter vector *V* for the T-S classifier. The specific learning steps are as follows:

- *Step 1*. The DTW-DFCM algorithm is used to divide the subclasses in each pattern class and determine typical signal samples in each. These subclasses form the kernel centers of each FRBN and determine the number of nodes in the FRBN layer.
- *Step 2*. FRBAIN training control parameters are set, and all parameters are initialized.
- *Step 3*. The output *o*_*j*_(*j*=1,2,…, *m*) of each FRBN is calculated for the input signal samples *x*_1_(*t*), *x*_2_(*t*),…, *x*_*n*_(*t*) using equation (3).
- *Step 4*. The FRBN layer outputs are regularized.
- *Step 5*. The outputs of each node in the rule layer are calculated using equation (6), and connections are established between the rule and regularization layers.
- *Step 6*. Fuzzy classifier outputs are calculated using equation (7).
- *Step 7*. The gradient descent algorithm is used to learn all FRBAIN parameters.

## 4. Experiment and Analysis

### 4.1. The Datasets

The data used in this experiment consisted of 12-lead ECG signal samples from the Chinese Cardiovascular Disease Database (CCDD). Each recording time was more than 10 seconds and included 9 heartbeats [35]. Each record lasted more than 10 seconds, including 9 heartbeats. The samples are marked with heartbeat segmentation and the diagnosis results by medical experts. Atrial premature beats, frequent premature beats, atrial tachycardia, and atrial fibrillation with rapid ventricular rate exhibit similar distributions and complex combination characteristics. In addition, the number of samples available for different disease types varied significantly. Experimental data consisted of 926 atrial premature beat, 985 frequent premature beat, 408 atrial fibrillation with rapid ventricular rate, and 389 atrial tachycardia samples, selected to form a small-scale and imbalanced database with 2708 signals.

### 4.2. The FRAIN Model for ECG Signal Classification

In the experiment, the DTW-DFCM algorithm was used to cluster a sample set of 4 diseases. The cluster numbers for the atrial premature beats, frequent premature beats, atrial tachycardia, and atrial fibrillation with rapid ventricular rate were 5, 6, 4, and 5, respectively. There were 20 pattern subclasses clustered in total. The clustering centers of these subclasses were selected as typical feature signal samples, and each pattern subclass corresponded to the fuzzy set of 4 diseases.

Network structure parameters in the E-FRAIN model, shown in Figure 3, were set with 12 nodes in the input layer, 20 nodes in the FRBN layer, 4 nodes in the pattern layer, 4^4^=256 nodes in the rule layer, and 256 nodes in the regularization layer II. The T-S fuzzy classifier included 256 input nodes and 1 output node. The stochastic gradient descent algorithm with Adam optimizer is used to train the model parameters. The training set samples are divided into 50 batches, each batch has 54 samples, which are trained in batches. Every 50 training cycles, the learning rate will be adjusted to 1/10 of the previous batch. The initial learning rate was set at 0.5. The maximum number of iterations is 500, and the final learning rate is 0.005. When the training error is less than 0.005, the training ends.

### 4.3. Experimental Results and Analysis

#### 4.3.1. Experimental Analysis

The sample set was randomly divided into 2 groups according to the proportion of illnesses, of which 1800 samples constituted the training set and the remaining 908 samples formed the test set. Property parameters and E-FRAIN connection weights were determined using the learning algorithm discussed in Section 3. Training error accuracy was set to 0.05, the maximum number of iterations was 3000, and the learning efficiency was 0.25. An overall accuracy rate of 87.56% was achieved in classifying test set samples. Corresponding evaluation indexes are shown in Table 1.

As seen in the table, the classification results achieved by the proposed technique are comparable to those of existing algorithms. This is because feature knowledge for typical signal samples, based on pattern subclasses, was embedded in the E-FRBAIN to effectively establish the model structural and data constraints. This approach also had the effect of reducing model parameters, which improved robustness for modeling small-scale and imbalanced sample sets. The membership degree for pattern subclasses was also used as an information unit to improve the model's identification capabilities for complex signal features, thereby maintaining the diversity of pattern features.

### 4.4. A Comparative Experiment and Analysis

In the comparative experiment, three types of deep neural network models were selected to directly classify multi-channel process signals. This included the multichannel deep convolutional neural network (MC-DCNN) [10], an algorithm combining LSTM with random forest (LSTM + RF) [36], and the deep gated recurrent unit (GRU) recurrent network (GRU-RNN) [37]. The same training and test sets were applied to each.

The architecture of the MC-DCNN model used in this experiment was *I* − *C*1(Size) − *S*1 − *C*2(Size) − *S*2 − *H* − *O*, where “Size” denotes the kernel size, *C*1 and *C*2 denote the number of filters, and *S*1 and *S*2 denote subsampling factors. The terms *I*, *H*, and *O*, respectively, represent the number of input layers, units in the hidden layer, and units in the MLP output layer. A comparative analysis suggested an architecture of 12-8(5)-2-4(5)-2-440-4 to be optimal. The LSTM + RF model used in the experiment was constructed using a series model of two LSTM networks, with 3 hidden layers in each LSTM. A random forest classifier with 100 trees was established in the feature vector space used for classification. The deep GRU recurrent network was superimposed with 5 GRU units and included a Softmax classifier.

A 4-fold crossover method was used in the experiment. The sample set was randomly divided into 4 groups according to the disease proportion, with 677 samples in each group. Three of these were combined to form the training set and one group was used as the test set. Four experiments were performed and the average value of each evaluation index in the experimental results was used as the comparison index. These results are shown in Table 2, where it is evident the proposed technique achieved the best results across all evaluation indicators.

This is because the model embeds diverse pattern class feature knowledge of signal samples in the mechanism. Decisions were then based on a fuzzy set of pattern subclasses. The structural and data constraints are implemented, and the number of model parameters is reduced, which improved signal feature identification ability and generalization. The other models are end-to-end deep learning algorithms for time-series signals, which include more parameters. In the case of incomplete and small-scale imbalanced datasets, the model structure and parameter selection include large degrees of freedom, which can result in overfitting and decreased generalizability. In addition, compared with other comparison methods, the time complexity and training time of proposed method in this paper have been greatly increased, mainly due to the time cost on iterative use of the DTW algorithm in training, but the average precision has been greatly improved.

The average correct recognition rate and mean standard deviations and *t*-test [38] were used as performance evaluation contrast index in the experiments, and the results are shown in Table 3.

In the experiment, the proposed method achieves good results in both the training set and the test set. The three other deep learning models have achieved good results in the training set learning, but the performance index and generalization property of the test sets are greatly reduced.

Comprehensive analysis shows that compared with general fuzzy neural network, the proposed method has advantages in feature knowledge embedding and fuzziness. It can express the features of signal samples in fine granularity, keep the diversity of features, and reduce semantic adhesion. Compared with it, the learning properties and generalization ability of deep neural networks have a strong dependence on the completeness of the dataset. For large-scale complete datasets, deep neural networks have advantages. However, in the case of small-scale imbalanced datasets, the deep neural network models have more parameters and large degrees of freedom, and the features of the pattern class with less samples are often weakened and suppressed during training, and the recognition accuracy and generalization ability are unstable. Due to the embedding of diversity prior feature knowledge, the proposed method can achieve the structural and data constraints, can improve the robustness and generalization ability of the model, and has good adaptability to modeling of small-scale incomplete datasets in the mechanism.

## 5. Conclusion

A fuzzy radial basis adaptive inference network was proposed in this study, which embeds prior feature knowledge for pattern classes in mechanism, effectively realized structural and data constraints of the model, and improved the modeling properties of small-scale imbalanced datasets. The membership functions for fuzzy sets, fuzzy inference rules, and classification rules could be determined adaptively, based on sample set learning. Due to the DTW-DFCM algorithm used to cluster and divide the pattern subclasses of each pattern class, the number of nodes in each layer can be computable, so that the FRBAIN can be regarded as a deterministic model. Simultaneously, the inference and classification of whole network are based on membership degree information from fuzzy sets, so that the FRBAIN exhibits both fuzziness and randomness. These bring convenience to the practical application of the model and better generalization properties and robustness. Based on the construction of radial basis fuzzification layer and the feature embedding mechanism of fuzzy radial basis neuron, it is convenient to embed the new typical feature knowledge of pattern class, expand and maintain the model, and improve the recognition ability of signal features. The comparative experiments results show that in the case of small-scale imbalanced datasets, the recognition rate of this method is 5.37% higher than other methods in the experiment, and other performance evaluation indicators are also significantly improved. The proposed method has good applicability in small-scale dataset modeling, but for large-scale datasets without obvious statistical characteristics, the computational complexity will increase exponentially. In addition, it has a strong dependence on the selection of typical feature samples, and has higher requirements for the similarity measurement of time-varying signal distribution characteristics, and the workload of selecting diverse typical feature samples in each pattern class is also relatively large. The proposed method can be extended to the field of typical feature embedding in pattern recognition, attention mechanism in image detection and segmentation, model architecture construction in multimodal data integration analysis, and so on, to achieve the structural and data constraint of the model. It has great application potential and value for research in unknown or low-cognition fields.

## Figures and Tables

**Table 1:** Experimental results for the proposed technique.

Type	Precision	Recall rate	*F*1-score
Atrial premature beat	0.9012	0.9419	0.9211
Frequent ventricular premature beat	0.9194	0.8636	0.8906
Atrial tachycardia	0.7632	0.8467	0.8028
Atrial fibrillation with rapid ventricular rate	0.8361	0.7786	0.8063

**Table 2:** A comparison of ECG signal classification results for various models.

Model	Precision (%)	Recall rate (%)	*F*1-score (%)	Training time (min)
MC-DCNN	78.73	79.34	79.03	11
LSTM + RF	80.19	81.22	80.70	21
GRU-RNN	77.93	78.68	78.30	14
Proposed	85.50	85.77	85.63	37

**Table 3:** A comparison of ECG signal classification results for various models.

No.	Model	Training set		Test set		*t*-test (*p* value)
		Average accuracy (%)	Mean standard deviations	Average accuracy (%)	Mean standard deviations	
1	MC-DCNN	98.32	0.0294	75.93	0.1227	0.0891
2	LSTM + RT	97.69	0.0326	81.21	0.0914	0.0855
3	GRU-RNN	98.22	0.0341	79.53	0.0756	0.0646
4	Proposed	98.47	0.0368	83.42	0.0473	0.0512

## Data Availability

The data used to support the findings of this study are included within the article.

## References

[1] Bououden S., Chadli M., Karimi H. R. (2015). Control of uncertain highly nonlinear biological process based on Takagi–Sugeno fuzzy models. *Signal Processing*.

[2] Yin S., Shi P., Yang H. (2015). Adaptive fuzzy control of strict-feedback nonlinear time-delay systems with unmodeled dynamics. *IEEE Transactions on Cybernetics*.

[3] Hussien M. A., Mahmoud T. A., Mahmoud M. I. (2017). Dynamic recurrent neural network based indirect adaptive control for nonlinear systems. *Menoufia Journal of Electronic Engineering Research*.

[4] Gao J., Zhang T., Xu C. A unified personalized video recommendation via dynamic recurrent neural networks.

[5] Derya Übeyli E. (2010). Recurrent neural networks employing Lyapunov exponents for analysis of ECG signals. *Expert Systems with Applications*.

[6] Tu K., Cui P., Wang X., Yu P. S., Zhu W. Deep recursive network embedding with regular equivalence.

[7] Karim F., Majumdar S., Darabi H., Chen S. (2017). LSTM fully convolutional networks for time series classification. *IEEE Access*.

[8] Yang J., Nguyen M. N., San P. P., Li X., Krishnaswamy S. (2015). Deep convolutional neural networks on multichannel time series for human activity recognition. *IJCAI*.

[9] Zhao B., Lu H., Chen S., Liu J., Wu D. (2017). Convolutional neural networks for time series classification. *Journal of Systems Engineering and Electronics*.

[10] Zheng Y., Liu Q., Chen E., Ge Y., Zhao J. L. Time series classification using multi-channels deep convolutional neural networks.

[11] Shiqian Wu S., Meng Joo Er M. J., Yang Gao Y. (2001). A fast approach for automatic generation of fuzzy rules by generalized dynamic fuzzy neural networks. *IEEE Transactions on Fuzzy Systems*.

[12] Uyar K., İlhan A. (2017). Diagnosis of heart disease using genetic algorithm based trained recurrent fuzzy neural networks. *Procedia Computer Science*.

[13] Fei J., Wang T. (2019). Adaptive fuzzy-neural-network based on RBFNN control for active power filter. *International Journal of Machine Learning and Cybernetics*.

[14] Camastra F., Ciaramella A., Giovannelli V. (2015). A fuzzy decision system for genetically modified plant environmental risk assessment using Mamdani inference. *Expert Systems with Applications*.

[15] Liu Y.-T., Wu S.-L., Chou K.-P. Driving fatigue prediction with pre-event electroencephalography (EEG) via a recurrent fuzzy neural network.

[16] Nazari S., Fallah M., Kazemipoor H., Salehipour A. (2018). A fuzzy inference-fuzzy analytic hierarchy process-based clinical decision support system for diagnosis of heart diseases. *Expert Systems with Applications*.

[17] Ilbahar E., Karaşan A., Cebi S., Kahraman C. (2018). A novel approach to risk assessment for occupational health and safety using pythagorean fuzzy ahp & fuzzy inference system. *Safety Science*.

[18] Mohamad D., Mukhtar F. L. (2018). Weighted Mamdani-type fuzzy inference system based on relative ideal preference system. *Journal of Soft Computing and Decision Support Systems*.

[19] Kazeminezhad M., Etemad-Shahidi A., Mousavi S. (2005). Application of fuzzy inference system in the prediction of wave parameters. *Ocean Engineering*.

[20] Huang D.-s. (1999). Radial basis probabilistic neural networks: model and application. *International Journal of Pattern Recognition and Artificial Intelligence*.

[21] Karayiannis N. B., Mi G. W. (1997). Growing radial basis neural networks: merging supervised and unsupervised learning with network growth techniques. *IEEE Transactions on Neural Networks*.

[22] Spooner J. T., Passino K. M. (1999). Decentralized adaptive control of nonlinear systems using radial basis neural networks. *IEEE Transactions on Automatic Control*.

[23] Xu S., He X. (2004). Research and applications of radial basis process neural networks. *Journal of Beijing University of Aeronautics and Astronautics*.

[24] Kong L., He W., Yang C., Li Z., Sun C. (2019). Adaptive fuzzy control for coordinated multiple robots with constraint using impedance learning. *IEEE Transactions on Cybernetics*.

[25] He W., Wang T., He X., Yang L.-J., Kaynak O. (2020). Dynamical modeling and boundary vibration control of a rigid-flexible wing system. *IEEE/ASME Transactions on Mechatronics*.

[26] He W., Mu X., Zhang L., Zou Y. (2020). Modeling and trajectory tracking control for flapping-wing micro aerial vehicles. *IEEE/CAA Journal of Automatica Sinica*.

[27] Cook A., Mısırlı G., Fan Z. Anomaly detection for iot time-series data: a survey. *IEEE Internet of Things Journal*.

[28] Chandra S., Gaur P. (2020). Radial basis function neural network technique for efficient maximum power point tracking in solar photo-voltaic system. *Procedia Computer Science*.

[29] Li H., Liu J., Yang Z., Liu R. W., Wu K., Wan Y. (2020). Adaptively constrained dynamic time warping for time series classification and clustering. *Information Sciences*.

[30] Munusamy S., Murugesan P. (2020). Modified dynamic fuzzy C-means clustering algorithm-application in dynamic customer segmentation. *Applied Intelligence*.

[31] Berndt D. J., Clifford J. Using dynamic time warping to find patterns in time series.

[32] Müller M. (2007). Dynamic time warping. *Information Retrieval for Music and Motion*.

[33] Bezdek J. C., Ehrlich R., Full W. (1984). FCM: the fuzzy c-means clustering algorithm. *Computers & Geosciences*.

[34] Zhu L., Wang J.-S., Wang H. (2019). A novel clustering validity function of FCM clustering algorithm. *IEEE Access*.

[35] Zhang J.-w., Liu X., Dong J. (2012). CCDD: an enhanced standard ECG database with its management and annotation tools. *International Journal on Artificial Intelligence Tools*.

[36] Sharma L. D., Sunkaria R. K. (2018). Inferior myocardial infarction detection using stationary wavelet transform and machine learning approach. *Signal, Image and Video Processing*.

[37] Rajan D., Thiagarajan J. J. A generative modeling approach to limited channel ECG classification.

[38] Nadeau C., Bengio Y. (2003). Inference for the generalization error. *Machine Learning*.

